# Process of Development of a County-wide Crisis Care Plan – Riverside County, California, 2016-7

**DOI:** 10.1371/currents.dis.f272fef04c7222a546e03450221a69d1

**Published:** 2018-10-01

**Authors:** Cameron Kaiser, Ramon Leon, Karen Craven

**Affiliations:** Department of Public Health, Riverside University Health System, Riverside, CA, USA; Emergency Management Department, County of Riverside, Riverside, CA, USA; Department of Public Health, Riverside University Health System, Riverside, CA, USA; Emergency Management Department, County of Riverside, Riverside, CA, USA

## Abstract

Introduction: Disasters with substantial impacts to the health care and public health systems can have multiple reverberating effects, including the need to alter the medical standard of care as well as centrally control scarce medical resources. A current crisis care plan can help to establish an ethical and operational framework for stakeholders before such a disaster takes place. However, there are few examples of such a plan that cover large areas and health jurisdictions. This article describes the process of developing such a “Crisis Care Plan.”

Methods: Plan developers from the Riverside County Department of Public Health and Riverside County Emergency Management Department first developed an ethical framework for decision making, followed by the development of a full operational crisis care plan with conditions for activation, life cycle and deactivation. The plan was then reviewed by major county stakeholders, including local emergency medical services, the county medical association and the hospital association, and additional comments incorporated. Before the final plan is implemented it will be submitted for public review and provider training materials will be developed.

Results: The development of a prerequisite ethical framework helped to reduce the risk that the operational plan would cause or exacerbate care disparities by informing a blinded, objective process for evaluating resource requests centrally prior to distribution. The ethical framework served to establish the grounding principle of all lives having an equal claim on value. Stakeholders recognized the need for such a Crisis Care Plan and agreed with the underlying ethical principles. Stakeholders also contributed useful recommendations to enable the plan to operate in as successful a manner as possible under the difficult conditions within which it would exist.

Discussion: The development of a clear ethical framework and the early identification and involvement of stakeholders can enable even very large health jurisdictions to construct crisis care plans that enable the best care under difficult circumstances, while protecting individual rights and incorporating the concerns of the public and the health care community.

## Introduction

The impact of a major disaster upon local health care and public health systems can have multiple cascading effects. Events such as Hurricanes Katrina and Harvey can cause widespread damage and disruptions to normal operations that reverberate long after the immediate crisis has passed. Maintaining a functioning healthcare system during and in the immediate aftermath of a large scale disaster requires consideration of issues such as equitable distribution of scarce resources, sudden changes to the standard of medical care and the moral and ethical ramifications that may ensue when crisis measures must be implemented.^1^

As a means towards improved community resiliency and faster and more organized recovery, disaster and crisis care plans can help to establish ethical guidelines^2^ and protocols and procedures before such events occur so that inequities are minimized, care as close to the standard is maximized, and stakeholder acceptance is achieved.^3^ However, there have been few attempts to devise and implement crisis care plans at the local governmental level and fewer examples still that document the methods and processes undertaken. This article describes the process taken by the County of Riverside, California to construct its own crisis care plan, presently the 10th largest county in the United States by population, and to document the manner, methods, successes and challenges encountered during its design and systemization.

## Background

Riverside County, California, with its county seat of Riverside approximately 60 miles east of Los Angeles, was established in 1893 as one of the state’s 58 counties. It is presided over by a five-member Board of Supervisors and serviced by an almost 25,000-employee municipal workforce. With a population of over 2.3 million^4^ it extends east from San Diego and Orange counties all the way to the Colorado River and the Arizona state line for 7,208 land square miles.

Its large rural areas, 17 general acute-care hospitals, 52 skilled nursing homes and significant numbers of residents below the federal poverty level present unique challenges to local readiness efforts. Existing hazard analyses of the County show that the vast majority of presidential disaster declarations have come from flooding, almost three times as many as from wildfires.^5^ Wildfires remain a chronic threat due to the arid climate of Southern California, however, as well as earthquakes due to the presence of the infamous San Andreas Fault and numerous smaller seismic faults throughout. The county’s large population size and proximity to Los Angeles also put it at risk of pandemics and bioterrorism, either as a direct target or spreading eastward from the Los Angeles basin.^6^

Under county and state statute, emergency declarations can be unilaterally declared by a number of county officials, including the public health officer, the California equivalent of the public health commissioner, which under the California Health and Safety Code is the legal authority charged with sanitation and containment of communicable disease.^7^ Such declarations must be ratified by the Board of Supervisors within one week. During an emergency declaration, the health officer “may take any preventive measures that may be necessary to protect and preserve the public health” which can include, but is by no means limited, necessary adjustments to the local medical standard of care and determining sites where such care may be administered.^7^ Inherent in this power is the decision-making capacity to determine what requests shall be prioritized when health resources are limited or impacted, which is exerted through the Medical Health Operational Area Coordinator program jointly operated by the health officer and the director of the emergency medical services agency,^8^ a branch of the county emergency management department. This operational authority was key in the construction of the actual plan.

## Determining the Plan Framework

County officials recognized the need for a crisis care plan several years prior to this plan’s completion. In 2016, the public health officer met with staff from the county emergency management department to discuss its implementation. After completion of an initial draft by the public health officer, a team consisting of the health officer, the chief of the public health emergency preparedness and response branch, and the branch’s preparedness program manager met for further review and discussion.

The need to pre-establish a robust ethical framework for the crisis care plan was determined very early in its conception to make sure critical care was delivered and distributed equitably, and that we would not exacerbate existing disparities in a crisis situation. Most of the available literature emphasized quantifying need and benefit as a means to make the process impartial and quantitative, as well as the imperative to preserve equitability, patient autonomy and the beneficence of the system within any response^9^^, ^10^, ^11 and the ethical responsibility of physicians and public health professionals to “steward resources during a period of true scarcity.”^10^ The concept of beneficence was especially considered to ensure that it could be maintained even if life-preserving resources could not be provided; in such cases palliative care and non-abandonment could satisfy this requirement,^12^ but only if the process were shown to be transparent and fair to stakeholders and the public.^10^

Procedurally, multiple studies identified the Sequential Organ Failure Assessment (SOFA) Scoring System^13^ as a means of triage,^9^^, ^14 which combines rapid assessment of key organ and physiologic functions into a single score predictive of mortality that can be serially assessed. However, there was also wide consensus that SOFA and similar assessment methods should not be the only means of ranking utility, as opposed to simply survivability, so as not to discriminate against those with preexisting medical issues^14^ or cause exacerbation of preexisting disparities in the community.^15^ At least one published source advised augmenting decisions with net-benefit calculations such as total lives saved, finding this view “widely accepted during a public health emergency” since it incorporates greater intrinsic equity by giving each life “an equal claim on being saved.”^12^ Whatever decisions are made, they should be supported by sound data, nondiscriminatory, sensitive to the needs of vulnerable populations, and revisable, with transparency, stakeholder participation and clear accountability.^15^

With an eye to similar ethical principles, our literature search suggested four specific operational principles: an “equitable triage process” using SOFA scores; triage by senior clinicians that do not have direct clinical obligations, with a support system to manage the triage process; legal and ethical constructs that underpin the allocation of scarce resources; and a mechanism for revision of the process (even, potentially, during a single disaster scenario) as further information becomes available.^9^ While few sources attempted to address activation, possibly due to concerns over generalization, potential triggers could include undeliverable or inaccessible health care resources, multiple facilities being impacted, limited (or an expectation that there will be limited) access to medical care resources, and/or the depletion of resource caches without short-term resupply.^1^^, ^16

## Constructing the Plan

Based on these perspectives, our team selected the principles of minimum disturbance (implementation of crisis care plans should only be under extraordinary circumstances, for the minimum period required, and for only the impacted resources); preservation of individual patient autonomy and respect, even if the patient does not receive the affected resource(s); stakeholder involvement at every phase, from plan construction to activation and after-action evaluation; the recognition of every life having an equal claim of value and that the plan should maximize lives saved; and that no decision is final, especially in the face of continually changing situational awareness. A working example plan was then constructed with both this framework and identified best practices in mind, to be activated in a declared public health emergency impacting multiple facilities with a critical shortage of a lifesaving aid unlikely to be relieved in the near term. The key sections and components of the plan are depicted in Table 1.

**Table 1: Key sections of the Crisis Care Plan.** Key sections and subsections within the proposed county-wide crisis care plan.

Section	Subsections
Mission and purpose statements	
Authorities and definitions	Enumerating authority for initiating and operationalizing the plan
	Functions of public health officials
Communications plan	Continuity with existing medical/health department operations plan
	Integration with Incident Command System communication pathway
Concept of operations	Literature review
	Ethical framework and key ethical principles
	Planning assumptions and criteria for activation
	Life cycle for activation, operational periods, and deactivation
	Decision-making process and guidelines for clinical review team
Annexes	Threat and hazard model
	Organizational charts for Incident Command System
	Community training plan

Our first draft plan was envisioned to operate in continuity with the existing county medical surge plan as an overlay upon the county’s standardized resource request pathway. Appropriate authority was identified within state and local ordinance for activation of the process by the public health officer through the Medical Health Operational Area Coordinator program. Upon activation, the plan envisioned a Crisis Care Clinical Review Team notionally placed under the Planning Section of the county’s Incident Command System structure assembled of the health officer, and the medical directors for the emergency medical services agency and the fire department, as depicted in Figure 1.

**Incident Command System organizational chart with Crisis Care Clinical Review Team, prior to community review.**
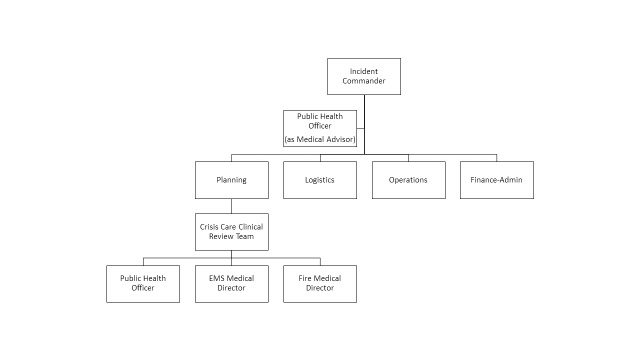

The review team would be tasked with enumerating affected resources and determining the documentation necessary to evaluate requests for those resources (such as history and physical examination documentation, admission notes, progress notes, flowsheets and laboratory or diagnostic results; we also selected the SOFA as a means of quantitative triage scoring). During operational periods, the review team would convene at least once to review incoming requests, redisposition requests that were deferred or denied, and determine if the crisis care plan needed to remain in effect or be modified or terminated as situational awareness evolved. This initial draft process is depicted in Figure 2.

**Crisis Care Plan theory of operations, prior to community review.**
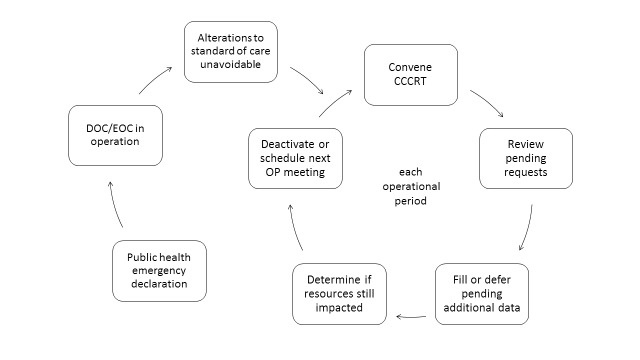

With a complete internal first draft, we began the stakeholder participation and adoption phase by determining three tiers of community stakeholders: internal stakeholders within the county public health system; organizational-level stakeholders outside of the county public health system; and physicians, nurses and health systems. We also added the director of the public health department, a separate position from the public health officer, as well as the director of the county emergency management department to our team of internal stakeholders.

Once internal stakeholders had signed off on the process, we moved to organizational-level stakeholders outside of the categorical county public health system. Our key goals with this second tier were to gauge overall acceptance of our approach, learn early on about areas of concern from these stakeholders as well as the medical providers and health systems they represent, and ensure that our plan appropriately reflected their probable functional level during such an incident. To that end, after multiple discussions, we selected representatives from the local medical association, the hospital association and the county sheriff, as well as the director of the local emergency medical services agency and a member of the EMS agency’s executive staff.

This expanded second tier understood clearly the goals of the plan, appreciated their role in this early stage of involvement, and were broadly supportive of both the ethical framework and the plan itself. They made several recommendations, including adding additional community physician members to the clinical review team; the revision is shown in Figure 3.

**Incident Command System organizational chart with Crisis Care Clinical Review Team, after review by second tier.**
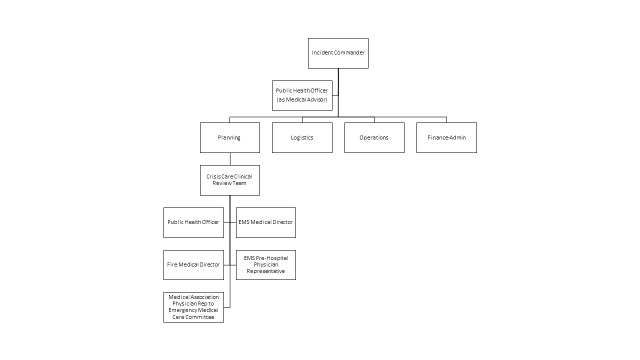

The second tier also advised changing the frequency of triage requests in order to streamline the process of acquiring data and simplify decision-making for both the requesting facility and the emergency operations center. These suggestions were incorporated into a new draft of the plan, key elements of which appear in Table 1, and this updated version was brought into alignment with the county medical surge plan. This revised pathway with stakeholder input is shown in Figure 4.

**Crisis Care Plan theory of operations, after review by second tier.**
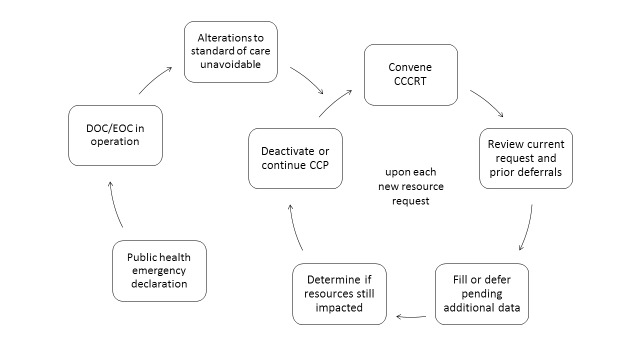

This second group also met and discussed how to disseminate the plan to the third tier, the greater physician and health system community, through a public comment process and potential working groups or trainings. These plans are currently in the process of being finalized as part of an upcoming systemwide readiness exercise and are expected to be an ongoing process.

## Discussion and Lessons Learned

There are few previous examples of local crisis and disaster care plans, and fewer still for local governments with large populations. Although the limited literature on this issue impairs our ability to analyze why there is such a paucity, our experience with our local plan suggests that procedural aspects and the wide variety of potential stakeholders tend to be the two greatest barriers for developing such a plan. Even though we used what we thought was a particularly expansive definition to decide whom to involve, new stakeholders who sometimes initially appeared only peripherally related were added to our list as the plan development progressed. This became a complicating factor because substantial discussion occurred repeatedly with both internal and external stockholders about the circumstances under which the plan would take effect, the extent of its authority, who should participate in decision-making, how often resources decisions should be made, and under what circumstances its mandate would continue and terminate. As the stakeholder pool increased, the work required to reach consensus also rose exponentially; an opportunity for improvement would have been to identify as many stakeholders as possible as early as possible. In addition, such discussions can be controversial, especially when potentially affected individual providers feel their medical authority is being usurped or that their clinical decisions are being second-guessed, or that hospital procedure is being circumvented. The impact of such a crisis on local public safety departments and emergency medical services agencies also gives rise to concerns of their own over security. Civil disorder in the wake of potential life-saving resources being rationed and transported and the possibility of altered standards for resuscitation in the field could put first responders in jeopardy. All of these concerns emerged and were discussed at length in our stakeholder meetings, and we expect them to be points of discussion for our readiness exercises as well.

Unfortunately, there is rarely a one-size-fits all solution for a local jurisdiction attempting to construct such a plan; every jurisdiction has its own unique physical, political, demographic and budgetary realities. We did learn, however, that the most effective way to reach consensus on controversial aspects of the plan was to maintain an open, inclusive and transparent development process. The involvement of stakeholders early on, and an expansive definition of what constitutes a stakeholder, were important factors in building trust and confidence in the development process and reassuring our stakeholders that their concerns were being heard and respected. We strove greatly to observe the maxim that, when the plan affects the greater community, the community gets a vote. We also made sure that our planning processes were flexible and, other than the basic ethical framework which virtually every stakeholder concurred with, not tied to demanding a particular process outcome or a preconceived notion about how such a plan should operate.

We also learned in our literature search that the medical science was far from settled as to individual prognosis when evaluating a resource request. Although objective survivability assessments would appear to help avoid obvious biases, not only can we never be fully certain that such assessments do not themselves have biases that can reinforce existing disparities, but we must also consider the gestalt of the entire request and keep clearly in mind that all lives have an equal claim on value. Even as our stakeholders appreciated keeping the process as objective, population-oriented and metrics-based as possible, it was still important to their acceptance to enable reviewers to consider the individual perspective and recognize that objective diagnostic values may not tell the entire story.

We certainly hope never to have a disaster of this magnitude in Riverside County that would require activation of the crisis care plan, and while we feel confident in that our plan is the best possible product from a long and sometimes arduous process, it can never be known if it will work as intended in advance. What has certainly become clear to us, however, is that it was necessary and expedient to have embarked upon the process; after all, one can never answer the questions that are never asked. It is quite unfortunately likely that some place, somewhere, will suffer a severe shock to their own health infrastructure sometime in the very near future, and we hope our experiences are instructive to other jurisdictions in planning for such a situation.

## Data Availability Statement

The full text of the Crisis Care Plan, as well as all supporting figures, related plans and documentation, are available by contacting the corresponding author, Cameron Kaiser, at ckaiser@rivcocha.org, or via the County of Riverside Department of Public Health at http://www.rivcoph.org/.
